# Supplementary material for: Inverse Association between Statin Use and Cancer Mortality Relates to Cholesterol Level
Source: Cancers (Basel). 2022 Jun 14;14(12):2920. doi: 10.3390/cancers14122920 (PMC9221017; doi:10.3390/cancers14122920)
Supplement: Supplementary file 1 [file cancers-14-02920-s001.zip › cancers-1759683-supplementary.pdf]

Supplementary table S1. Risk of cancer death by statin use with or without adjustment for total cholesterol level in the Finnish Randomized Study of Screening for Prostate Cancer.

	Risk of cancer death by statin use	Risk of cancer death by statin use with further adjustment for total cholesterol level
	HR (95% CI)	HR (95% CI)
Gastric cancer	0.79 (0.47-1.33)	0.89 (0.52-1.53)
Liver cancer	0.54 (0.34-0.88)	0.66 (0.41-1.08)
Non-Hodgkin lymphoma	1.36 (0.79-2.37)	1.57 (0.89-2.77)
Renal cancer	1.84 (1.00-3.41)	2.46 (1.31-4.63)
Bladder cancer	0.86 (0.47-1.58)	1.03 (0.55-1.90)
Central nervous system cancer	1.19 (0.63-2.26)	1.23 (0.63-2.39)
